# Efficacy of Cardiac Computed Tomography for Obstructive Mechanical Valve Thrombosis Under Venoarterial Extracorporeal Membrane Oxygenation

**DOI:** 10.1016/j.jaccas.2022.09.011

**Published:** 2022-11-16

**Authors:** Wenhann Chen, Kensuke Yokoi, Goro Yoshioka, Keiji Kamohara, Koichi Node

**Affiliations:** a Department of Cardiovascular Medicine, Saga University, Saga, Japan; b Department of Thoracic and Cardiovascular Surgery, Saga University, Saga, Japan

**Keywords:** cardiac computed tomography, obstructive mechanical valve thrombosis, venoarterial extracorporeal membrane oxygenation, CT, computed tomography, VA-ECMO, venoarterial-extracorporeal membrane oxygenation

## Abstract

We encountered a patient in a state of shock who required venoarterial extracorporeal membrane oxygenation in whom cardiac computed tomography was instrumental in diagnosing obstructive mechanical mitral valve thrombosis as well as in the differentiation of other probable diseases. Because the patient was on venoarterial extracorporeal membrane oxygenation support, computed tomography imaging required some ingenuity. (**Level of Difficulty: Intermediate.**)

## Central Illustration

**Figure:**
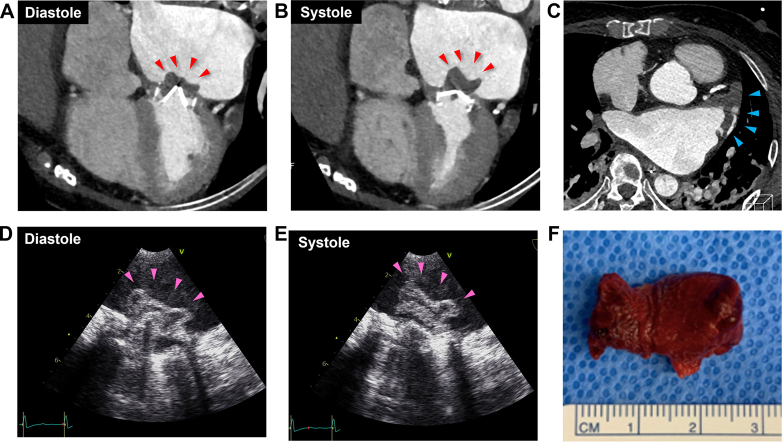

A 62-year-old Japanese woman was admitted to our hospital for surgical excision of a parotid gland tumor. She had a surgical mechanical valve replacement 16 years prior and chronic atrial fibrillation. She was routinely administered warfarin, which was bridged with intravenous unfractured heparin 1 week before the surgery. Three days after the surgery, the patient suddenly developed chest pain and went into extreme shock. Because there were no signs of infection or bleeding and her echocardiography showed reduced left ventricular wall motion, she was considered to be in cardiogenic shock. Venoarterial-extracorporeal membrane oxygenation (VA-ECMO) was initiated in the catheter laboratory to stabilize her circulation. The cause of perioperative cardiogenic shock requires differentiation of multiple diseases.^1^

**Learning Objectives**

• Cardiac CT images are comprehensible to even non cardiologists, aiding in the diagnosis of fulminant obstructive mechanical valve thrombosis.
• Cardiac CT is useful for investigating the cause of cardiogenic shock requiring VA ECMO, by careful adjustment of the contrast medium injection, scan timing, and flow rate of VA-ECMO.

In the catheter laboratory, acute myocardial infarction was excluded by using coronary angiography. Fluoroscopy of the mechanical mitral valve indicated marginally reduced mobility of the 2 leaflets, with no difference between them, refuting a definitive diagnosis of obstructive mechanical mitral valve thrombosis. Transesophageal echocardiography is the most frequently recommended option for further investigation of obstructive mechanical mitral valve thrombosis^2^; however, we needed to exclude other causes of cardiogenic shock, including pulmonary embolism and acute aortic dissection. Therefore, electrocardiogram-gated cardiac computed tomography (CT) was performed within 1 hour after VA-ECMO initiation.

Hemodynamic changes in patients on VA-ECMO can cause inconsistent mixing of the flow from the native heart and incomplete opacification of the heart chambers, which can result in inaccurate CT images.^3^ Ingenuity is required for CT to overcome these pitfalls. On VA-ECMO support, it was difficult to predict when the pulmonary artery and left atrium would be filled with the contrast medium. Therefore, to prolong the duration, the contrast medium was injected at 2 mL/s for 60 s instead of the usual rate of 5 mL/s. Using a retrospective ECG-gated helical dual-source CT scan, images were taken separately for each of the 2 phases with a fixed delay time, as the pulmonary artery and left atrium were filled with contrast. To minimize the flow of the injected contrast agent into the VA-ECMO circuit, the flow rate was temporarily lowered from 3.5 L/min to 1.0 L/min while carefully ensuring that the circulatory dynamics did not deteriorate and that they returned to the original setting after the injection was completed.

**Figure 1:**
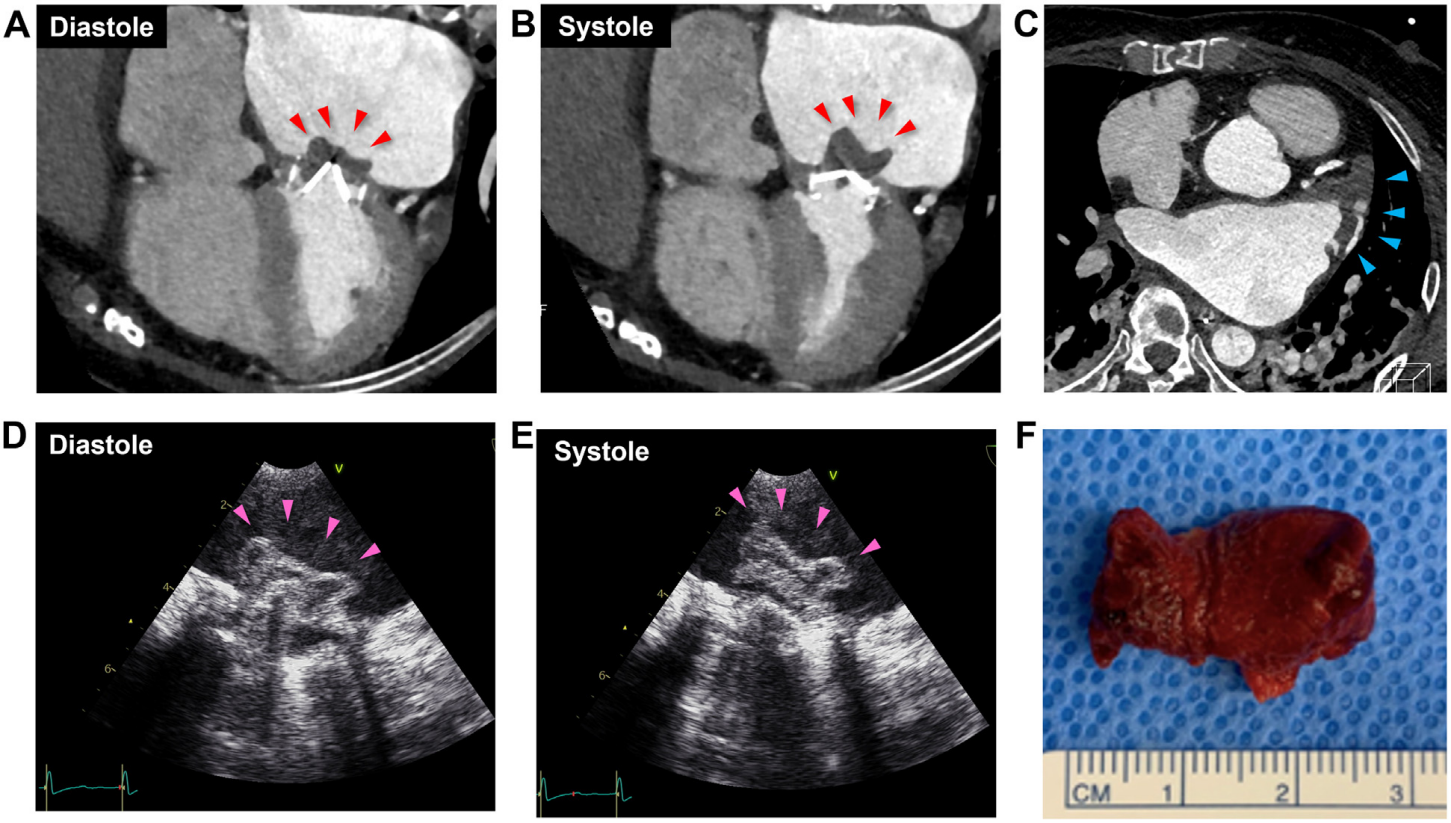
Cardiac Computed Tomography, Transesophageal Echocardiography, and Surgically Removed Thrombus **(A, B)** Cardiac computed tomography (CT) showing that the massive thrombus was attached to the mechanical mitral valve, which was mobile at the time of cardiac contraction (red arrowheads). **(C)** A thrombus in the left atrial appendage is also confirmed (blue arrowheads). **(D, E)** Transesophageal echocardiography confirmed the similar findings of the thrombus (pink arrowheads) shown in the cardiac CT. **(F)** The surgically removed thrombus specimen (2.5 × 1.5 cm) from the mechanical mitral valve.

The CT images revealed a mobile thrombus around the mechanical mitral valve prosthesis (Figures 1A and 1B, Video 1) and a left atrial appendage thrombus (Figure 1C). Pulmonary embolism or acute aortic dissection were then ruled out.

After the patient was transferred to the intensive care unit, we performed transesophageal echocardiography, which confirmed similar thrombus findings (Figures 1D and 1E, Video 2).

The patient underwent a surgical thrombectomy and left atrial appendage plication, and the 2.5 × 1.5 cm-sized thrombus was removed from the mechanical mitral valve (Figure 1F). The patient’s postoperative course progressed without further complications.

As demonstrated in this case, cardiac CT images are comprehensible to noncardiologists, aiding the diagnosis of fulminant obstructive mechanical valve thrombosis.

## Funding Support and Author Disclosures

The authors have reported that they have no relationships relevant to the contents of this paper to disclose.

## Appendix

Supplemental Video 1 Cardiac Computed Tomography

Supplemental Video 2 Transesophageal Echocardiography
